# A structural bioinformatics framework for prioritizing pH-sensitive proteins from 3D structural features

**DOI:** 10.1186/s12859-026-06415-1

**Published:** 2026-04-26

**Authors:** Amirhossein Akbarpour Arsanjani, Ziba Veisi Malekshahi, Bashir Mosayyebi, Babak Negahdari, Masoumeh Amirlou, Fatemeh Khavari, Davood Rabiei Faradonbeh

**Affiliations:** Department of Medical Biotechnology, School of Advanced Technologies in Medicine, Tehran University of Medical Sciences, Tehran, Iran

**Keywords:** Tumor microenvironment, PH-sensitive proteins, Protonation prediction, PROPKA, Structural bioinformatics

## Abstract

**Background:**

Acidic extracellular pH is a defining feature of many solid tumors and can influence the structural stability and activity of proteins involved in cancer progression. However, no unified computational framework exists for systematically assessing the pH-sensitivity of proteins using structural information.

**Methods:**

We developed a structural bioinformatics framework that quantifies pH-sensitivity by integrating five protonation-relevant descriptors derived from protein sequences and 3D structures: total histidine count, histidine proportion, ionizable residues with predicted pKa values in acidic ranges, solvent-exposed histidines, and helix-associated histidines. pKa values were obtained using PROPKA 3.0, and all feature extraction steps were implemented through custom Python scripts. The framework was applied to cancer-associated secretory and membrane proteins to prioritize those whose structures may be particularly susceptible to acidic tumor conditions.

**Results:**

The framework effectively ranked proteins according to their structural susceptibility to acidic pH and revealed distinct distributions of pH-sensitive features across secretory and membrane classes. Several high-scoring proteins, including ITGB8, LGR5, FZD7, HLA-E, ANGPTL2, and BST2 emerged as candidates potentially affected by protonation-linked perturbations in acidic tumor microenvironments. Enrichment analyses of ranked subsets demonstrated how score-derived protein groups can support downstream biological interpretation.

**Conclusion:**

This study presents a reproducible and extensible structural scoring framework and applies it to the systematic prioritization of cancer-associated proteins potentially vulnerable to acidic tumor pH. By combining 3D structural descriptors with protonation predictions, the approach offers a computational tool for investigating pH-dependent protein behavior in cancer biology and other contexts where local acidity shapes molecular function.

**Graphical Abstract:**

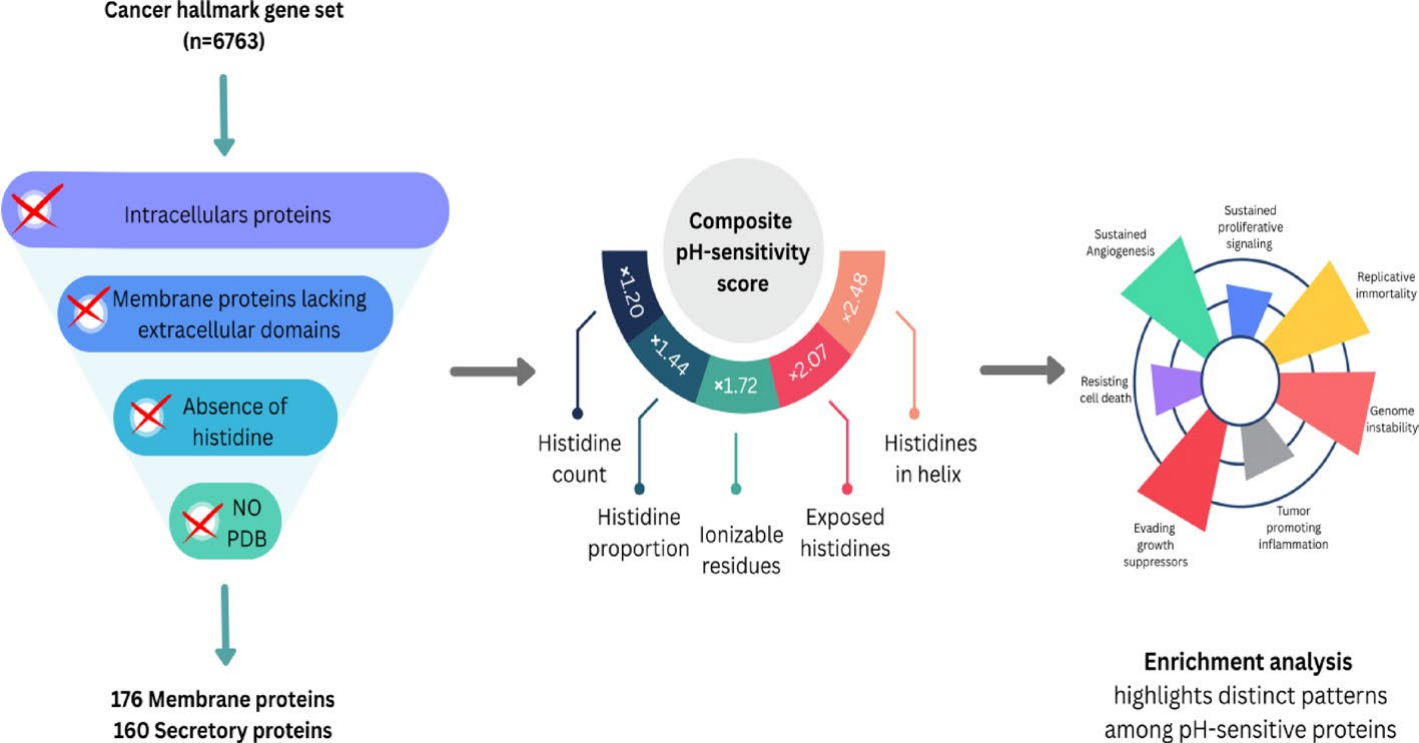

**Supplementary Information:**

The online version contains supplementary material available at 10.1186/s12859-026-06415-1.

## Introduction

 Acidic extracellular pH is a defining feature of many solid tumors and influences diverse processes such as proliferation, invasion, and immune evasion [1]. Tumor acidity arises through multiple metabolic adaptations, including elevated glycolysis, lactate export, carbonic anhydrase activity, and CO_2_ generating pathways that collectively promote proton accumulation in the extracellular space [2–4]. While intracellular acidification is cytotoxic, cancer cells maintain near-neutral intracellular pH through proton efflux systems, thereby establishing an inverted pH gradient (pHe < pHi) that distinguishes the tumor microenvironment from normal tissue [5–7]. In healthy tissues, the extracellular fluid is maintained within a remarkably narrow range of 7.40 ± 0.02. In contrast, the interstitial space of solid tumors frequently exhibits pH values ranging from 6.4 to 7.0 [8]. This altered physicochemical niche contributes to tumor progression, therapeutic resistance, and the emergence of aggressive cellular phenotypes [9–12]. The functional consequences of tumor microenvironment (TME) acidification are mediated through changes in the protonation states of ionizable amino acid residues within proteins. The dysregulated pH of the TME enables specific cancer behaviors by altering the function of proteins whose activities or binding affinities are regulated within the narrow range of pathological pH dynamics. While many signaling proteins are regulated by pHi, extracellular and membrane-associated proteins are directly exposed to the acidic pHe, influencing processes such as adhesion, migration, and immune evasion [13].The susceptibility of a protein to pH-driven changes is fundamentally determined by the acid dissociation constant (pKa) of its constituent side chains [14–18]. Among the twenty standard amino acids, histidine stands as the most versatile pH sensor due to its imidazole side chain, which possesses a pKa near physiological neutral (~ 6.0 to 6.5). This proximity to the pH range of the TME allows histidine to switch between neutral, aromatic states and positively charged, hydrophilic states in response to subtle environmental fluctuations [14, 19]. Interactions with neighboring residues, burial within a hydrophobic core, or proximity to charged groups can shift the pKa of histidine by as much as four units, ranging from 2.0 to 9.0 [20, 21]. Consequently, the identification of truly pH-sensitive sites requires a nuanced understanding of structural context beyond simple sequence content.

Advancements in structural bioinformatics have led to the development of various tools designed to predict the pH sensitivity of proteins [22, 23]. Despite the plethora of tools available, a significant gap remains in the ability to systematically prioritize pH-sensitive proteins from large-scale proteomic data. Current strategies for identifying pH-sensitive candidates typically rely on single measures, such as total histidine content or a single pKa prediction. In fact, simple sequence-based counts of histidine do not distinguish between structural passengers and functional sensors. A histidine buried in a rigid hydrophobic core may be “electrostatically frustrated” but may never undergo a charge shift within the physiological range of the TME. Besides, specific secondary structures, such as alpha-helix N-terminal caps, are known to utilize histidines as pH-dependent switches. These motifs are frequently overlooked in global sequence-based scans [20, 22, 24, 25].

The structural bioinformatics framework developed in this context addresses these gaps by integrating multiple orthogonal structural features into a single, weighted prioritization score. This approach represents a shift from raw biophysical measurement to a systems-level prioritization strategy. By combining sequence-derived features (FASTA-based counts) with 3D structural descriptors (PROPKA-derived SASA and secondary structure association), the framework mitigates the “conformational noise” inherent in single static structures. This multi-descriptor approach allows for a more robust ranking of the proteome, identifying high-priority candidates for downstream biochemical validation and therapeutic targeting.

## Methods

### Data collection and preprocessing

**Fig. 1:**
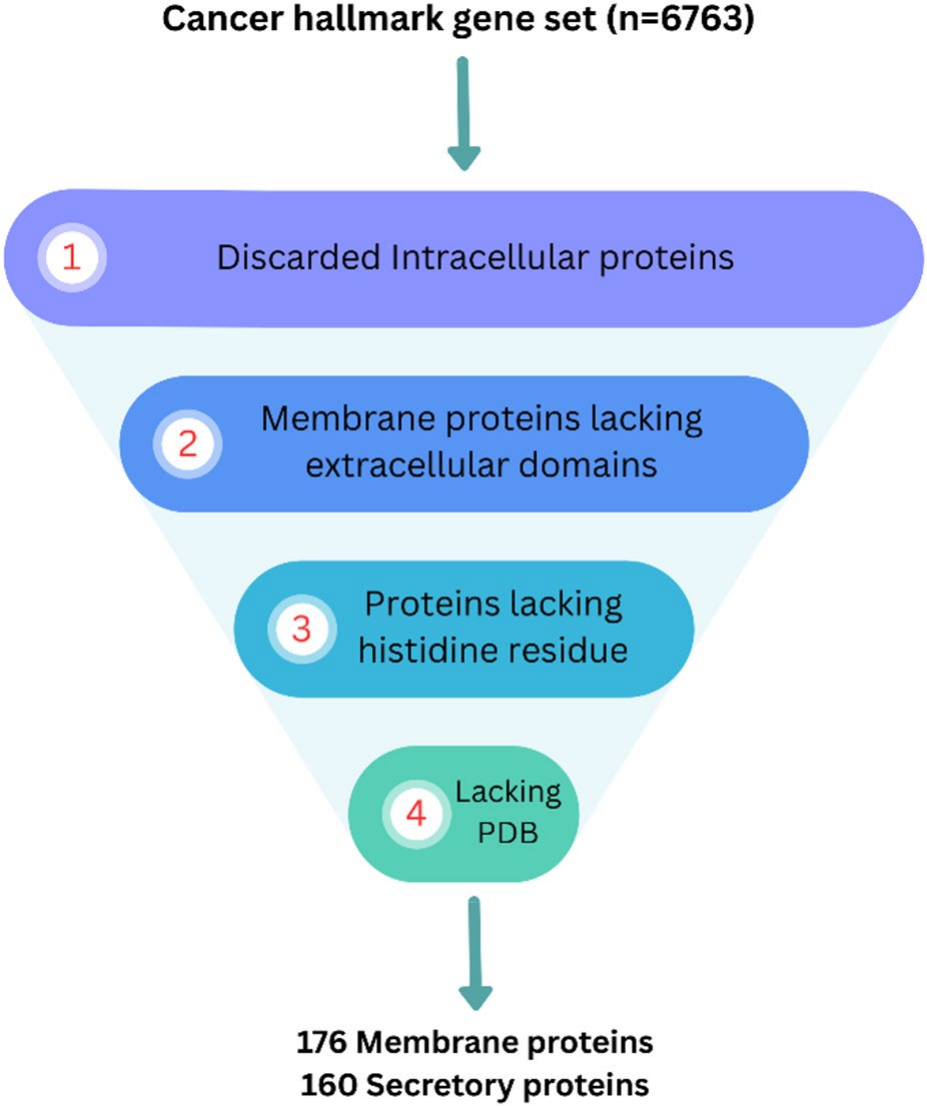
Dataset preprocessing and protein selection workflow. The initial cancer hallmark gene set comprised 6763 proteins. Sequential filtering steps were applied to exclude intracellular proteins, membrane proteins lacking extracellular domains, proteins without histidine residues, and proteins without available PDB structures. After these filtering steps, 336 proteins remained and were classified into two groups: 176 membrane proteins and 160 secretory proteins

We obtained cancer hallmark annotations from the CancerHallmarks.com resource (https://cancerhallmarks.com/download), which provides curated hallmark gene sets described in the associated reference publication [26]. Candidate proteins were included if they met the following criteria: (i) association with at least one cancer hallmark based on the CancerHallmarks.com annotations; (ii) availability of a corresponding amino acid sequence in UniProt and an experimentally determined 3D structure with a PDB entry; and (iii) presence of at least one histidine residue in the analyzed sequence region. Intracellular proteins and membrane proteins lacking extracellular domains were excluded. Proteins were classified into secretory or membrane-associated groups using UniProt subcellular localization annotations. For membrane proteins, only extracellular domains were retained for downstream analyses to focus on regions directly exposed to the acidic tumor microenvironment. Data preprocessing and downstream feature extraction were implemented in Python 3.10. The workflow of dataset preprocessing and protein selection is summarized in Fig. 1.

### Quantification of histidine content in sequences

Absolute and relative histidine content were computed for each protein. Amino acid sequences were retrieved in FASTA format from UniProt, and a custom Python script quantified (i) total histidine count and (ii) histidine percentage relative to sequence length. For membrane-associated proteins, calculations were restricted to extracellular residues.

### pKa estimation with PROPKA

Protonation behavior of ionizable residues was evaluated using PROPKA 3.0 [23, 27]. All structures were processed in batch mode through an automated shell–Python pipeline. From each PROPKA output, the SUMMARY section containing residue-level pKa values and solvent accessibility was extracted programmatically. Only ionizable residues with predicted pKa values in the range 6.0–7.2.0.2 (the physiologically relevant acidic-to-neutral interval characteristic of solid tumors) were retained. Residues outside this window were excluded, as they are unlikely to undergo protonation–deprotonation transitions in acidic tumor microenvironments.

### Identification of solvent-exposed histidines

Solvent accessibility of histidine residues was determined using the PROPKA-reported relative solvent accessible surface area (rSASA). Histidines with rSASA > 50% were classified as solvent-exposed, indicating a high likelihood of direct interaction with extracellular protons. For each structure, both the number and proportion of solvent-exposed histidines were recorded.

### Identification of helix-associated histidines

*α*-helices are the most abundant secondary structure and have fundamental roles in folding landscape and structure stability of proteins [28]. Histidine residues associated with *α*-helices are critical for modulating the stability of the native state and the transition state ensemble (TSE) [29]. Consequently, structural perturbations in helical regions are more likely to result in significant functional consequences compared to changes in disordered loops. When a histidine residue within an *α*-helix becomes protonated, it can profoundly impact the stability of the entire helix by interacting with the protein backbone charges [24]. Research indicates that charged histidines influence *α*-helix stability at all positions within the helix through these electrostatic interactions, often acting as specific pH-dependent switches such as those found in N-terminal caps [24, 25]. The structural context of a helix can influence the pKa of the histidine side chain, allowing it to transition between a neutral aromatic state and a positively charged hydrophilic state within the narrow, pathologically relevant pH range of the TME (6.4–7.0) [28].

Secondary structure annotations were obtained from HELIX records in PDB files. Helical segments were extracted based on their annotated residue ranges, and histidines within these extracted α-helical regions were counted to define helix-associated histidines. The total number of helix-associated histidines per structure was used as a structural descriptor of potential pH sensitivity. All scripts used in these analyses are provided in the Supplementary Files (Supplementary Code).

### Construction of a composite pH-sensitivity score and protein ranking

After extracting all sequence and structural-derived features (histidine count in FASTA, proportion of histidine in the FASTA, pKa-filtered ionizable residues, solvent-exposed histidines, and helix-associated histidines), we constructed a quantitative metric to estimate the overall pH-responsiveness potential of each protein. We reasoned those proteins exhibiting higher values across these features are more likely to undergo structural or functional changes in acidic tumor environments [25, 30–34].

To enable direct comparison across features with different scales, all feature values were normalized to a 0–1 range using min–max normalization:

$$P\_normalized=\frac{(P-P\_min)}{P\_max-P\_min}$$

Where P is the raw parameter value, and P_min and P_max represent the minimum and maximum observed values of that parameter, respectively.

Each feature was then assigned a weight reflecting its presumed biological relevance to pH sensitivity. Weighted contributions were calculated using the following formula:

$$ Wi = \frac{{k\left( {n - r_{i} + 1} \right)}}{{\mathop \sum \nolimits_{{j = 1}}^{n} k^{ \wedge } \left( {n~ - ~r_{j} ~ + ~1} \right)}} $$

Where k is a coefficient multiplier (set to 1.2), n is the number of parameters (*n* = 5), and r_i_ is the rank of the i-th parameter. The denominator encompasses all parameter ranks (rⱼ) and ensures that the resulting weights are normalized (i.e., the sum of all weights equals 1). This normalization step guarantees that the relative importance of each parameter is maintained in proportion to its assigned rank. In this framework, the five parameters were ranked according to their presumed biological relevance to pH-driven structural changes. Total histidine count was assigned the lowest importance, followed by histidine percentage, then the number of ionizable residues with pKa values between 6.0 and 7.2. Solvent-exposed histidines received a higher weight, and histidines located in α-helical segments were assigned the highest weight, reflecting the central role of surface-accessible, helix-associated histidines as potential pH-responsive structural motifs. The exponential weighting scheme therefore prioritizes features that are more likely to directly mediate conformational responses to acidic tumor microenvironments.

The final composite score (S) for each protein was computed as a weighted sum of the normalized values:

$$ S = \mathop \sum \limits_{{i = 1}}^{n} Wi.~\,P\,~normalized,i $$

This score reflects the relative likelihood of pH responsiveness for each protein. Proteins within each category (secretory and membrane proteins) were subsequently sorted in descending order based on their composite score. The ranked lists were used for downstream quartile-based functional analyses and hallmark enrichment comparisons.

The hierarchical ranking of these structural descriptors and the specific biophysical justifications for their assigned weights are summarized in Table 1.

**Table 1:** Structural descriptor ranking

Rank	Structural descriptor	Biological justification	Weighting class
1	Helix-associated Histidines	Dictates secondary structure transitions and stabilizes the transition state ensemble (TSE) via metal coordination or pH-dependent folding [29].	Highest Weight
2	Solvent-exposed Histidines	Primary mediator of pH-dependent binding due to high accessibility and predictable titration behavior [20].	High Weight
3	pKa-filtered Residues	Refines sensor selection by identifying specific residues undergoing significant ionization changes within the therapeutic pH range (6.0 to 7.4) [20].	Medium Weight
4	Histidine %	Serves as a global metric for the relative density of potential pH-responsive sites, providing a sequence-level baseline for overall sensitivity across the protein chain	Low Weight
5	Total Histidine Count	Represents the cumulative protonation potential of the primary sequence; included as a foundational indicator established in existing sequence-based screenings	Lowest Weight

### Pathway enrichment analysis of pH-sensitive proteins

To investigate the functional relevance of pH-sensitive proteins, we performed a pathway-level analysis using the Hallmarks of Cancer database [26]. Proteins were analyzed separately within the secretory and membrane groups. To compare the biological functions of highly versus less pH- sensitive proteins, we divided the ranked list into four quartiles (Q1–Q4). The top quartile (Q1), representing the most pH-sensitive proteins, was compared against the remaining three quartiles (Q2–Q4). This enrichment approach allowed us to explore whether proteins harboring protonation-sensitive features are preferentially involved in hallmark pathways like invasion and metastasis, angiogenesis, immune evasion, or proliferation.

### Statistical analysis

All statistical analyses were performed in Python 3.10 using NumPy, SciPy, and Pandas. All feature values were min–max normalized prior to composite score construction to ensure comparability across different biological metrics.

Proteins in the membrane and secretory groups were independently ranked according to their composite pH-sensitivity scores and stratified into quartiles (Q1-Q4). In the membrane group (*n* = 176), Q1 comprised 44 proteins, while the remaining 132 proteins were distributed across Q2-Q4. Similarly, in the secretory group (*n* = 166), Q1 included 40 proteins and remaining 120 proteins were assigned to Q2-Q4.

For hallmark enrichment analysis, the top quartile (Q1) was compared against the combined lower quartiles (Q2–Q4) based on the frequency of proteins annotated to each hallmark category.

Statistical significance of differences in hallmark representation was evaluated using chi-square (χ²) tests. To control for multiple comparisons, Benjamini–Hochberg false discovery rate (FDR) correction was applied. Adjusted p-values < 0.05 were considered statistically significant.

## Results

### Distribution patterns of composite scores reveal distinct levels of heterogeneity between protein groups

Composite scores were normalized independently within the secretory and membrane groups. Therefore, absolute score values are not directly comparable across groups. However, the shape and spread of the score distributions provide meaningful insight into the variability of pH-responsive structural features within each category. Secretory proteins displayed a narrow, tightly clustered distribution of composite scores, indicating relatively low variability and a more homogeneous set of pH-related structural characteristics. In contrast, membrane-associated proteins exhibited a broader distribution with a long right-tail, reflecting substantially greater heterogeneity in their pH-sensitivity related features. This wider spread suggests that extracellular domains of membrane proteins encompass a more diverse range of structural motifs-some highly enriched in pH-responsive features such as solvent-exposed or helix-associated histidines, and others much less so. The score distributions for secretory and membrane proteins are illustrated in Fig. 1.

### Top-ranked pH-sensitive proteins in membrane and secretory groups

**Fig. 2:**
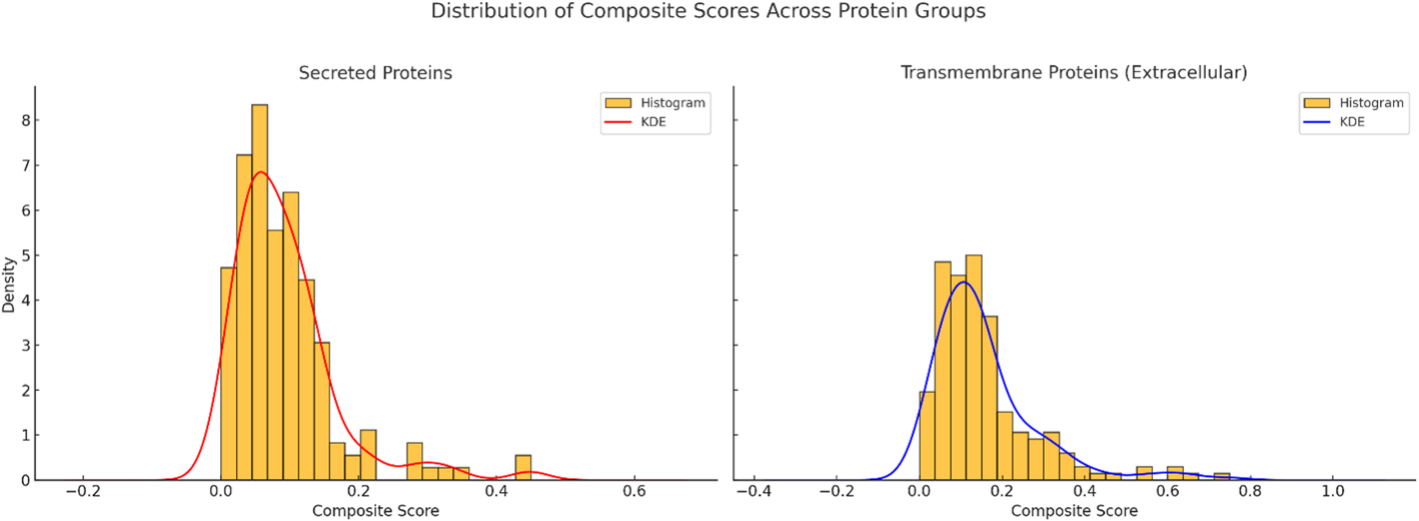
Distribution of composite scores calculated for secretory and membrane proteins. Histogram bars represent the frequency of proteins within score intervals, and KDE curves illustrate the overall distribution pattern in each group. Normalization performed separately for each group; distributions not directly comparable

The proteins with the highest composite pH-sensitivity scores in the membrane-associated and secretory groups are shown in Fig. 2. These top-ranked proteins represent candidates whose structural features collectively indicate a higher potential sensitivity to acidic pH conditions. The complete ranked lists for both protein groups, along with the values of all features contributing to the composite score, are provided in the Supplementary Files (Supplementary Data).

### Hallmark enrichment analysis highlights distinct functional patterns among highly pH-sensitive proteins

**Fig. 3:**
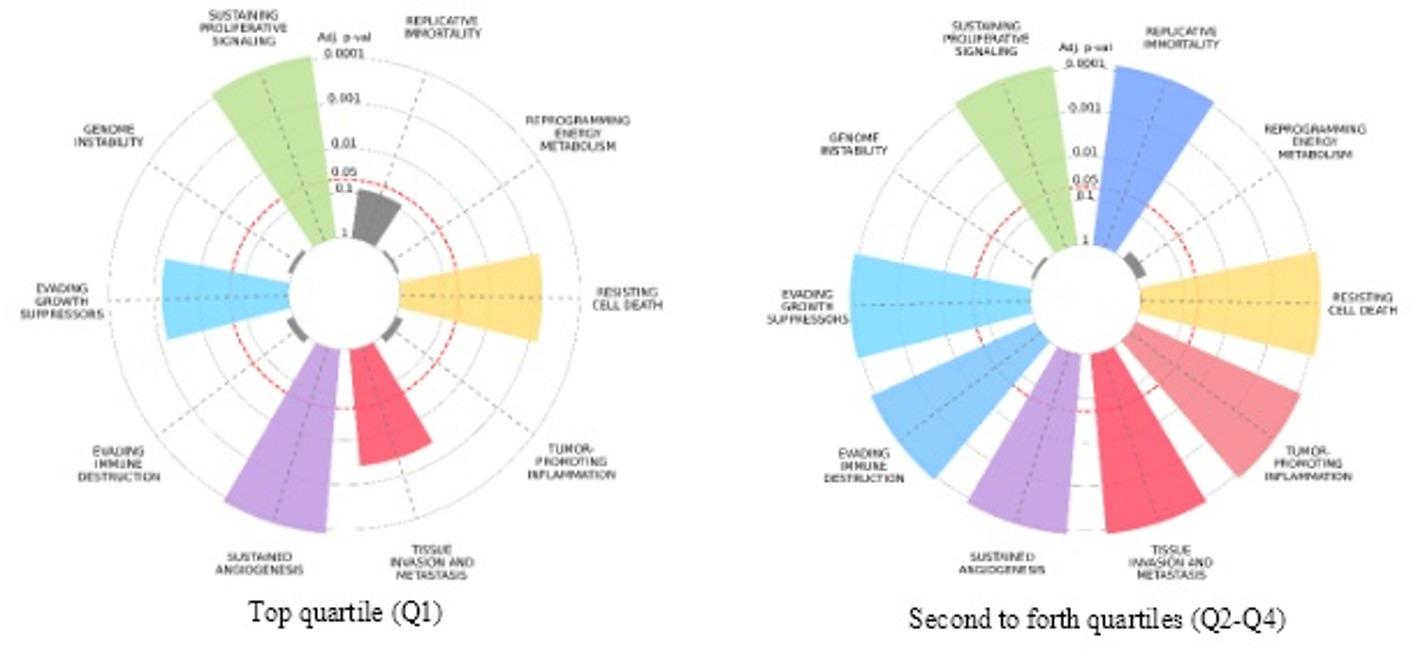
Comparative hallmark enrichment analysis for secretory proteins stratified by composite score. The analysis compares proteins in the top quartile (Q1, *n* = 40) representing the pH-sensitive candidates, against those in the combined second to fourth quartiles (Q2–Q4, *n* = 120). Bars represent the statistical significance (adjusted p-values) of associations across various cancer hallmark categories. The red dashed line indicates the threshold for statistical significance (adjusted *p* = 0.05)

**Fig. 4:**
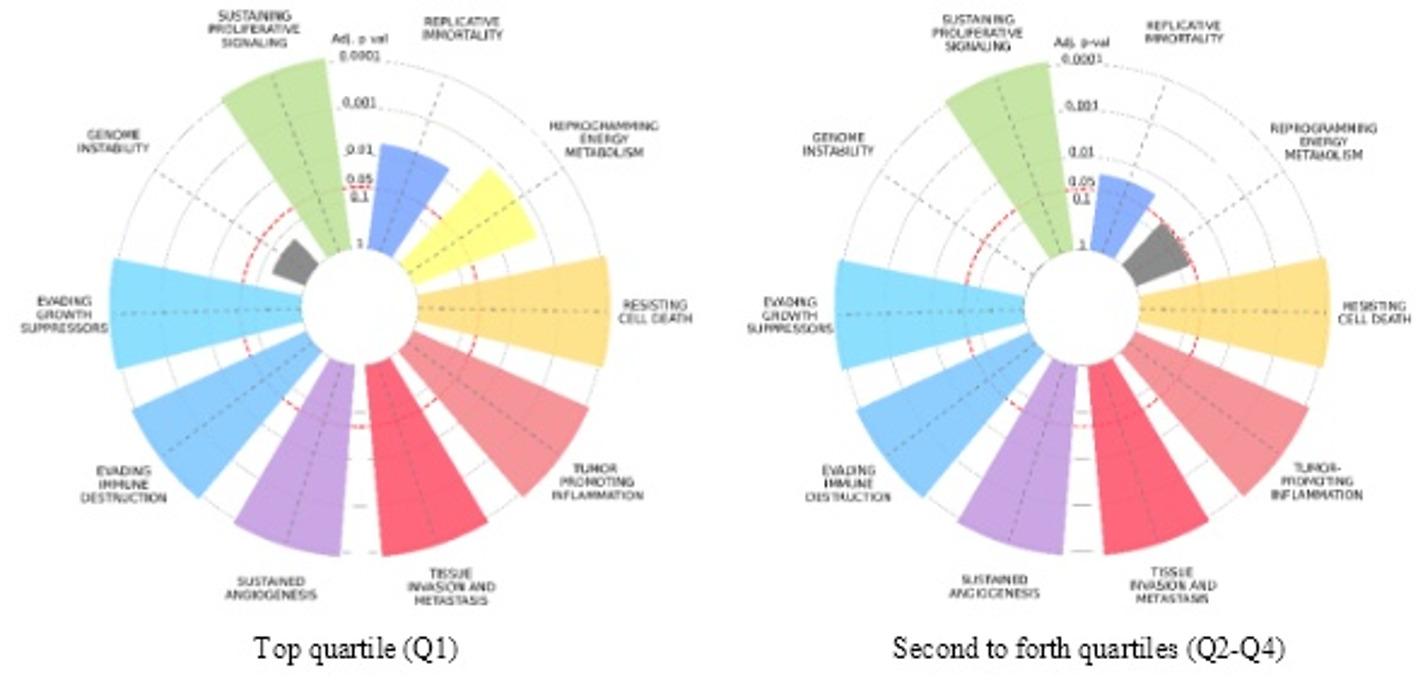
Comparative hallmark enrichment analysis for membrane proteins stratified by composite score. The left panel displays enrichment for proteins in the top quartile (Q1, *n* = 44), while the right panel shows results for the combined lower quartiles (Q2–Q4, *n* = 132). Bars represent the statistical significance (adjusted p-values) of associations across various cancer hallmark categories. The red dashed line indicates the threshold for statistical significance (adjusted *p* = 0.05)

To evaluate whether proteins with high pH-sensitivity scores are preferentially associated with specific oncogenic processes, we performed hallmark-based enrichment analysis on quartile-stratified protein groups. Within each category (secretory and membrane), the top quartile (Q1) was compared with the combined lower quartiles (Q2–Q4). Figures 3 and 4 summarize the enrichment profiles for secretory and membrane proteins, respectively.

**Fig. 5:**
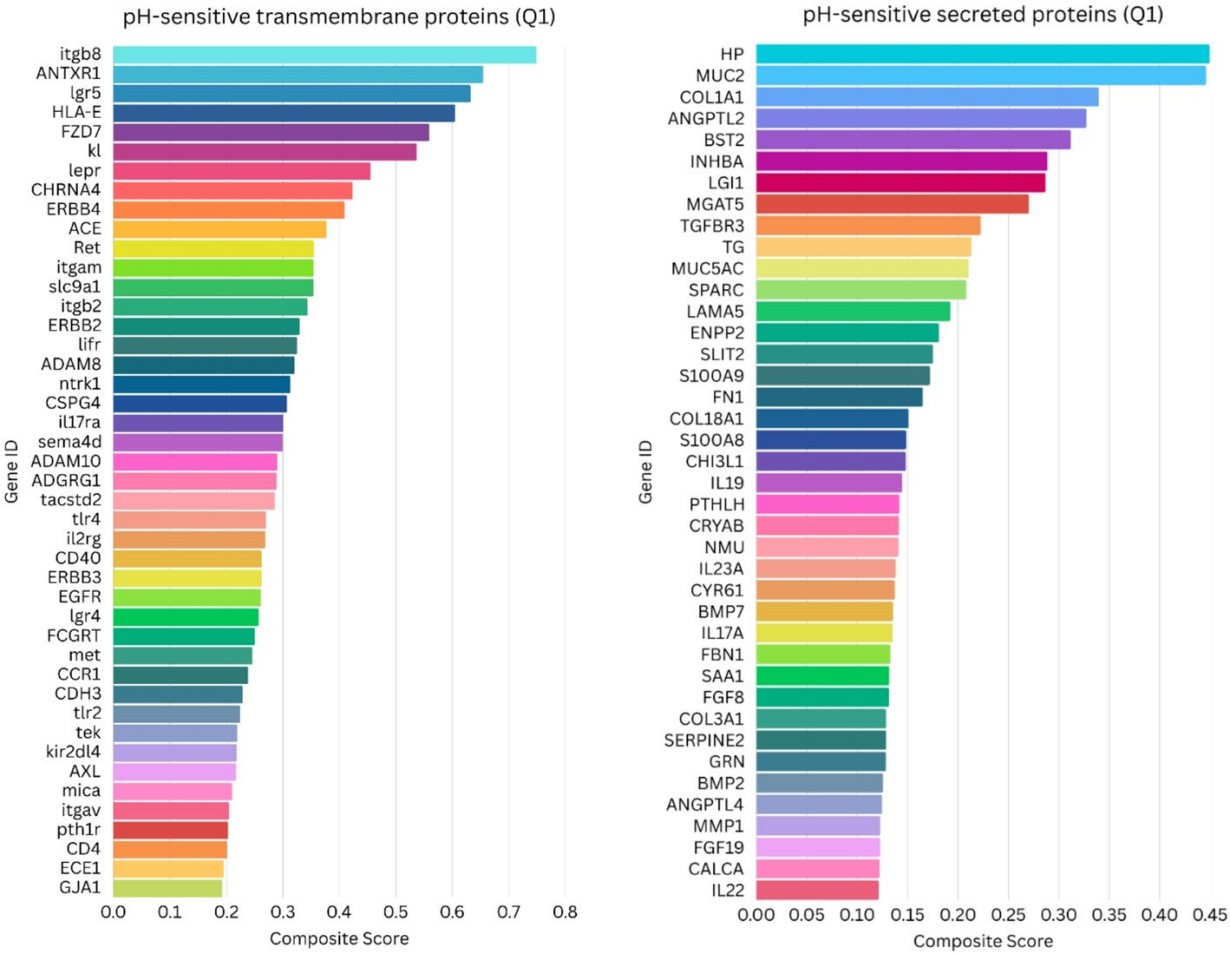
Top-ranked pH-sensitive proteins in membrane (left) and secretory (right) groups. Bars show composite pH-sensitivity scores for proteins in the top quartile (Q1) of each group, ranked from highest to lowest. Scores reflect a weighted integration of five structural and sequence-derived features: total histidine content, histidine percentage, number of ionizable residues within the 6.0–7.2 pKa window, solvent-exposed histidines, and helix-associated histidines. Complete ranked lists, including raw and normalized values for all five features, are provided in Supplementary files (Supplementary Data)

In the secretory protein group (Fig. 5), Q1 proteins exhibited selective enrichment for hallmarks such as sustaining proliferative signaling, resisting cell death, and sustained angiogenesis. In contrast, the Q2–Q4 subgroup displayed a more diffuse hallmark profile, with additional associations observed for replicative immortality, evading immune destruction, and tissue invasion and metastasis.

Table 2 summarizes the enrichment data for the top quartile (Q1) of the 160 identified secretory proteins.

**Table 2:** Hallmark enrichment for top-ranked (Q1) secretory proteins

Term	Overlap	*P*-value	Adjusted *P*-value
Sustaining Proliferative Signaling	22/3574	1.24E-05	5.56E-05
Genome Instability	0	nan	1
Evading Growth Suppressors	18/3288	0.000661003	0.001487256
Evading Immune Destruction	2/749	0.586644553	0.676456863
Sustained Angiogenesis	15/796	4.98E-10	4.48E-09
Tissue Invasion and Metastasis	14/2318	0.00140037	0.002520665
Tumor-Promoting Inflammation	2/769	0.60129499	0.676456863
Resisting Cell Death	14/1941	0.000224302	0.000672907
Reprogramming Energy Metabolism	1/740	0.861328535	0.861328535
Replicative Immortality	4/547	0.052622556	0.078933834

A similar yet distinct pattern emerged in the transmembrane protein group (Fig. 3). Q1 membrane proteins showed significant enrichment for proliferative signaling, resistance to cell death, and angiogenesis, paralleling the trends observed in secretory proteins, but also included prominent associations with pathways related to cell–cell communication and receptor-mediated signaling. In the Q2–Q4 subset, enrichment broadened to include hallmarks such as tumor-promoting inflammation, tissue invasion and metastasis, and evading immune destruction, reflecting the functional diversity of membrane-associated proteins with weaker pH-sensitivity signatures.

Table 3 summarizes the enrichment data for the top quartile (Q1) of the 176 identified membrane proteins.

**Table 3:** Hallmark enrichment for top-ranked (Q1) membrane-associated proteins

Term	Overlap	*P*-value	Adjusted *P*-value
Sustaining Proliferative Signaling	33/3574	1.08E-12	3.60E-12
Genome Instability	4/747	0.174520111	0.174520111
Evading Growth Suppressors	26/3288	1.01E-07	1.69E-07
Evading Immune Destruction	14/749	1.51E-08	3.02E-08
Sustained Angiogenesis	20/796	1.31E-14	1.31E-13
Tissue Invasion and Metastasis	28/2318	5.54E-13	2.77E-12
Tumor-Promoting Inflammation	13/769	1.81E-07	2.58E-07
Resisting Cell Death	22/1941	3.12E-09	7.80E-09
Reprogramming Energy Metabolism	8/740	0.001233686	0.001542107
Replicative Immortality	6/547	0.004972516	0.005525017

### Internal validation and ranking consistency analysis

To evaluate the refinement provided by the integrated scoring system, we performed a ranking consistency analysis. We compared the top-ranked proteins across three distinct prioritization criteria: total histidine count (our lowest-weighted sequence metric), helix-associated histidines (our highest-weighted structural metric), and the final composite score. This comparison serves as an internal benchmark to demonstrate how the inclusion of 3D structural context shifts prioritization away from simple sequence-level density toward high-priority functional motifs. In the membrane protein subgroup, significant shifts were observed between simple counts and the composite framework. As shown in Table 4, proteins such as ITGB8 and ANTXR1, which occupy the top two positions in the final composite ranking, were entirely absent from the top 10 list based solely on histidine count. This ranking shift suggests that while these proteins may not possess the highest absolute number of histidines, their residues are strategically positioned within helical segments or on solvent-exposed surfaces, making them superior candidates for functional pH-responsiveness. Conversely, a small subset of proteins, including kl and ACE, maintained high rankings across all three criteria, indicating they possess both high sequence-level potential and critical structural machinery.

A similar trend of structural refinement was evident in the secretory subgroup (see Table 5). While the total histidine count prioritized large, repetitive proteins like MUC5AC and LAMA5, the composite framework shifted the focus toward HP (Haptoglobin) and COL1A1, which are established in literature as having pH-sensitive properties [35, 36]. Notably, the top-ranked secretory proteins in the composite score showed a high degree of alignment with those ranked by helix-associated histidines, including BST2, ANGPTL2, and LGI1. These results collectively demonstrate that the composite score provides a significantly more refined prioritization than simple sequence counts.

**Table 4:** Ranking consistency for membrane proteins

Metric	Top 10 Proteins (Ranked 1 to 10)
Total Histidine Count	CSPG4, CR1, ADGRG1, CD40, ACE, CXCR4, kl, CD44, ADAM8,
Helix-Associated Histidines	ANTXR1, FZD7, ITGB8, ACE, HLA-E, kl, itgam, Ret, slc9a1, itgb2
Composite Score	ITGB8, ANTXR1, LGR5, HLA-E, FZD7, kl, lepr, CHRNA4, ERBB4, ACE

**Table 5:** Ranking consistency for secretory proteins

Metric	Top 10 Proteins (Ranked 1 to 10)
Total Histidine Count	MUC5AC, LAMA5, MUC2, LTBP1, FN1, FBN1, TG, COL18A1, SLIT2, ENPP2
Helix-Associated Histidines	HP, LGI1, BST2, ANGPTL2, TG, SPARC, SLIT2, ENPP2, NMU, CYR61
Composite Score	HP, COL1A1, ANGPTL2, BST2, INHBA, LGI1, MGAT5, TGFBR3, TGFBR3, TG

## Discussion

The acidic tumor microenvironment creates conditions under which extracellular and membrane-associated proteins may undergo protonation-driven structural alterations. In this study, we introduced a structural bioinformatics framework designed to quantify and rank the potential pH-sensitivity of proteins using a combination of sequence features, 3D structural descriptors, and PROPKA-derived protonation predictions. By integrating these heterogeneous features into a composite scoring system, the framework enables systematic identification of proteins whose conformational stability may be modulated by physiologically relevant acidic conditions.

Application of the framework to cancer-associated proteins revealed biologically meaningful patterns. Within the secretory protein group, several high-scoring candidates, such as HP, MUC2, COL1A1, ANGPTL2, and BST2 exhibited properties consistent with potential functional modulation under acidic conditions. Although our approach does not directly model structural rearrangements, prior biochemical evidence supports the plausibility of pH-dependent behavior in these proteins. For example, the interaction between haptoglobin and hemoglobin is known to exhibit pH-responsive characteristics, particularly in low-oxygen, acidic environments. Likewise, the polymerization and expansion behavior of MUC2 is strongly influenced by acidic-to-neutral transitions, and acidic conditions may stabilize more compact mucin assemblies capable of altering physical barriers in tumors. Collagen I, represented by COL1A1, is also susceptible to reduced tensile stability at mildly acidic pH, which may influence protease sensitivity and local matrix remodeling [35–37]. ANGPTL2 and BST2, both implicated in inflammatory and metastatic processes, also ranked highly. Although their precise pH-responsive mechanisms remain unclear, acidic stress is known to modulate secretory dynamics, extracellular vesicle composition, and membrane organization—processes in which these proteins participate. Thus, their elevated scores provide a hypothesis-generating perspective for future biochemical validation [38–40].

Among membrane-associated proteins, highly ranked candidates included ITGB8, ANTXR1, LGR5, HLA-E, and FZD7 proteins with roles in adhesion, receptor signaling, immune regulation, and stemness. Some of these proteins have documented pH-dependent behaviors. For instance, ANTXR1 participates in toxin binding events that occur at acidic pH, and integrins, including ITGB8, exhibit conformational landscapes sensitive to changes in protonation [41–43]. Proteins involved in Wnt signaling, such as LGR5 and FZD7, operate in microenvironments where extracellular ionic and pH conditions fluctuate, making protonation effects biologically plausible [44, 45].

Comparison of ranked subsets highlighted distinct patterns between secretory and membrane protein groups. For secretory proteins, the most pH-sensitive quartile was preferentially enriched for proliferative and angiogenic programs. In contrast, membrane proteins exhibited broader hallmark representation across quartiles, yet high-scoring proteins showed an enhanced association with metabolic rewiring pathways. These trends may reflect differences in selective pressures acting on secreted versus membrane proteins: secreted factors implicated in systemic or microenvironment-spanning processes may require structural robustness across diverse pH conditions, whereas membrane proteins inherently operate at interfaces where proton gradients fluctuate and may tolerate pH-responsive structural features.

### Methodological implications

The patterns observed in the biological interpretation illustrate how the scoring system can highlight proteins with potential pH-responsive architectures. Importantly, these findings demonstrate the utility of integrating multiple orthogonal structural features (rather than relying on single metrics such as histidine content) to derive a more holistic estimate of pH-sensitivity. The framework is generalizable and can be applied beyond cancer-associated proteins to any context in which local pH variation is biologically relevant.

### Limitations

This study is computational and predictive in nature. PROPKA-derived pKa values, while informative, do not fully capture conformational dynamics or environmental changes occurring in vivo. Our approach also does not explicitly model structural rearrangements under different pH conditions, nor does it incorporate molecular dynamics, mutagenesis data, or experimental measurements of stability. Furthermore, hallmark enrichment analyses serve as interpretive tools rather than evidence of causality.

### Future directions

Future work could integrate constant-pH molecular dynamics simulations, incorporate sequence-based pH-response predictors, or validate selected high-scoring proteins experimentally through biophysical characterization. Expanding the framework to predicted structures (e.g., AlphaFold models) or additional organisms could broaden its utility. The scoring strategy may also serve as a prioritization layer for designing pH-responsive therapeutics or for selecting proteins for deeper structure-function studies under controlled pH perturbations.

## Conclusion

In this study, we developed a structural bioinformatics framework that integrates sequence-derived features, 3D structural descriptors, and PROPKA-based protonation predictions to estimate the pH-sensitivity of proteins. When applied to cancer-associated secretory and membrane proteins, the framework revealed distinct patterns of potential pH responsiveness and identified subsets of proteins whose structural properties make them plausible candidates for modulation under acidic tumor conditions. These results demonstrate the value of combining multiple complementary descriptors to obtain a more comprehensive assessment of pH-sensitive architecture.

Although demonstrated here within the context of tumor acidity, the framework may be adapted to other biological settings in which local pH fluctuations influence protein behavior. Overall, this work provides a practical computational framework that can support the exploration of how microenvironmental acidity may shape protein structure and function and can serve as a basis for future hypothesis-driven studies.

## Supplementary Information

Supplementary Material 1.

Supplementary Material 2.

## Data Availability

All datasets used in this study are publicly available. Cancer hallmark annotations were obtained from the CancerHallmarks.com resource (https://cancerhallmarks.com/download). Protein sequences were retrieved from UniProt (https://www.uniprot.org), and experimentally determined protein structures were obtained from the Protein Data Bank (PDB; https://www.rcsb.org). Processed data generated in this study, including ranked lists of secretory and membrane-associated proteins with extracted feature values and composite scores, are provided in the Supplementary Files. All custom scripts used for the main analytical steps of the study are provided in the Supplementary Files (Supplementary Code).
